# Stat1 Phosphorylation Determines Ras Oncogenicity by Regulating p27^Kip1^

**DOI:** 10.1371/journal.pone.0003476

**Published:** 2008-10-22

**Authors:** Shuo Wang, Jennifer F. Raven, Joan E. Durbin, Antonis E. Koromilas

**Affiliations:** 1 Department of Oncology, Faculty of Medicine, McGill University, Montreal, Quebec, Canada; 2 Lady Davis Institute for Medical Research, Sir Mortimer B. Davis-Jewish General Hospital, Montreal, Quebec, Canada; 3 Columbus Children's Research Institute, Columbus, Ohio, United States of America; 4 Department of Pediatrics, The Ohio State University College of Medicine, Columbus, Ohio, United States of America; University of Hong Kong, China

## Abstract

Inactivation of p27^Kip1^ is implicated in tumorigenesis and has both prognostic and treatment-predictive values for many types of human cancer. The transcription factor Stat1 is essential for innate immunity and tumor immunosurveillance through its ability to act downstream of interferons. Herein, we demonstrate that Stat1 functions as a suppressor of Ras transformation independently of an interferon response. Inhibition of Ras transformation and tumorigenesis requires the phosphorylation of Stat1 at tyrosine 701 but is independent of Stat1 phosphorylation at serine 727. Stat1 induces p27^Kip1^ expression in Ras transformed cells at the transcriptional level through mechanisms that depend on Stat1 phosphorylation at tyrosine 701 and activation of Stat3. The tumor suppressor properties of Stat1 in Ras transformation are reversed by the inactivation of p27^Kip1^. Our work reveals a novel functional link between Stat1 and p27^Kip1^, which act in coordination to suppress the oncogenic properties of activated Ras. It also supports the notion that evaluation of Stat1 phosphorylation in human tumors may prove a reliable prognostic factor for patient outcome and a predictor of treatment response to anticancer therapies aimed at activating Stat1 and its downstream effectors.

## Introduction

The signal transducers and activators of transcription (Stats) are a family of cytoplasmic proteins that function as signal messengers and transcription factors involved in cellular responses induced by cytokines and growth factors [1], [2]. Stat1, the prototype of the family, is essential for innate immunity [2] and plays an important role in immune surveillance of tumors [3]. Specifically, Stat1 knockout (Stat1^−/−^) mice are highly susceptible to virus infection [4], [5] and more prone to the formation of tumors in response to carcinogens than normal mice [6]. Stat1 is also an important mediator of the anti-proliferative and pro-apoptotic functions of interferon-gamma (IFN-γ) and tumor necrosis factor-β (TNF-β) through its ability to upregulate caspase 1 and the cyclin dependent kinase (Cdk) inhibitor p21^Cip1^ [7]–[10]. At the molecular level, cytokines and growth factors induce Stat1 phosphorylation at tyrosine (Y) 701, which is essential for its homo-dimerization or hetero-dimerization with other Stats and binding to DNA [1], [2]. Tyrosine phosphorylation of Stat1 is mediated by cytokine receptor associated *Janus* tyrosine kinases (Jaks) as well as by receptor tyrosine kinases (RTKs) [2]. Phosphorylation of Stat1 at serine (S) 727 is mediated by various pathways and is required for the full induction of Stat1-dependent gene transactivation [11].

The *Cdkn1b* gene encodes for a 27 kDa protein (p27), which belongs to the Cip/Kip family of cyclin-dependent kinase inhibitors (CKIs) [12]. p27^Kip1^ acts in G_0_ and early G_1_ to inhibit cyclin-Cdk holoenzymes, particularly cyclin E-Cdk2, and impair cell cycle progression [12]. p27^Kip1^ levels decrease in response to mitogenic signaling thus permitting cell cycle progression and cell proliferation [12]. The human *Cdkn1b* gene is present on chromosome 12p13 and loss of one allele has been observed in a number of human malignancies [13]. Consistent with a tumor suppressor function, mice lacking one or both copies of the *Cdkn1b* gene have increased susceptibility to carcinogen-induced tumorigenesis [14]. p27^Kip1^ does not follow Knudson's classic “two-hit hypothesis” of tumor suppression because homozygous loss or silencing of the *Cdkn1b* locus in human tumors is extremely rare [13]. The *Cdkn1b* gene is rarely mutated in human cancers but decreased concentrations of p27^Kip1^ are implicated in human tumorigenesis [13]. There is an inverse correlation between p27^Kip1^ levels and prognosis in a variety of human cancers, including those of breast, colon, and prostate origin [13]. Expression of the *Cdkn1b* gene is regulated at the transcriptional, translational and post-translational levels. Transcription is controlled by several factors including Sp1 [15], Phox2a [16], members of the forkhead box (Fox) group of transcription factors [17] and Stat3 [18], [19]. Early findings provided evidence for a cell-cycle dependent regulation of *Cdkn1b* mRNA translation [20]. Subsequent studies found that translation of *Cdkn1b* mRNA is controlled by sequences within the 5′ untranslated region (5′ UTR) [21], [22] through a cap-independent mechanism [23] and the utilization of an internal ribosomal entry site (IRES) [24]. The post-translational control of p27^Kip1^ is fairly complex and involves phosphorylation, changes in subcellular distribution as well as proteasomal degradation [12].

The anti-tumor properties of Stat1 have mainly been linked to its function downstream of IFNs[3]. This prompted us to examine whether Stat1 plays a role in oncogenic signaling in the absence of an IFN effect. Herein, we present a novel functional link between Stat1, p27^Kip1^ and oncogenic Ras. We demonstrate that Stat1 subverts the inactivation of p27^Kip1^ in Ras transformed cells by positively regulating the transcription of the *Cdkn1b* gene. We further demonstrate that induction of p27^Kip1^ expression by Stat1 is essential for the suppression of Ras-mediated oncogenesis *in vitro* and *in vivo* via mechanisms that are affected by the phosphorylation of Stat1 at tyrosine 701 and serine 727.

## Results

### Stat1 counteracts the downregulation of p27^Kip1^ by activated Ras

**Figure 1:**
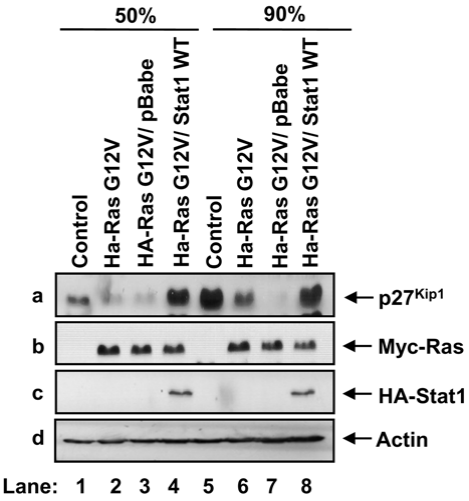
Stat1 prevents the decrease of p27^Kip1^ by activated Ras. Primary p53^−/−^Stat1^−/−^ MEFs (lanes 1 and 5) were transfected with Myc-Ha-Ras G12V (lanes 2 and 6) and reconstituted with HA-Stat1 WT by retrovirus infection (lanes 4 and 8). As control, Myc-Ha-Ras G12V expressing MEFs infected with empty retroviruses were used (lanes 3 and 7). Polyclonal populations were harvested at 50% (lanes 1–4) or 90% confluence (lanes 4–8) and cell extracts (50 µg of protein) were subjected to immunoblotting with anti-p27^Kip1^ monoclonal antibody (mAb) (panel a), anti-Myc mAb (panel b), anti-Stat1α mAb (panel c ) and anti-actin mAb (panel d).

To examine the role of Stat1 in Ras transformation, we used primary mouse embryonic fibroblasts (MEFs) from Stat1 and p53 double-knock out animals (p53^−/−^Stat1^−/−^ MEFs). We chose p53 deficient MEFs because p53 inactivation facilitates transformation by expression of cytoplasmic oncoproteins including activated Ras [25]. When primary p53^−/−^ Stat1^−/−^ MEFs were transfected with a Myc-tagged form of Ha-RasG12V, we noted that activated Ras decreased p27^Kip1^ levels as previously described [26] (Fig. 1, panel a, compare lane 1 with 2, and lane 5 with 6). The expression of p27^Kip1^ was affected by the density of the cells since p27^Kip1^ levels were proportional to the confluency of control cells (panel a, compare lane 1 with 5) and downregulation of p27^Kip1^ by activated Ras was more obvious in confluent than in sub-confluent cell cultures (Fig. 1, panel a, compare lanes 1 and 2 with 5 and 6). When the MEFs transfected with activated Ras were reconstituted with an HA-tagged form of human wild type (WT) Stat1 by retrovirus infection, we observed that Stat1 restored p27^Kip1^ protein levels in both sub-confluent and confluent cell cultures (panel a, lanes 4 and 8). Contrary to this, p27^Kip1^ levels remained low in MEFs infected with empty retroviruses (panel a, lanes 3 and 7). The expression of activated Ras (lane b) and reconstituted Stat1 (lane c) was not affected by the density of the cells as verified by immunoblotting. These data provided evidence that Stat1 subverts the donwregulation of p27^Kip1^ by activated Ras.

### p27^Kip1^ expression is controlled by phosphorylated Stat1 at the transcriptional level

**Figure 2 pone-0003476-g002:**
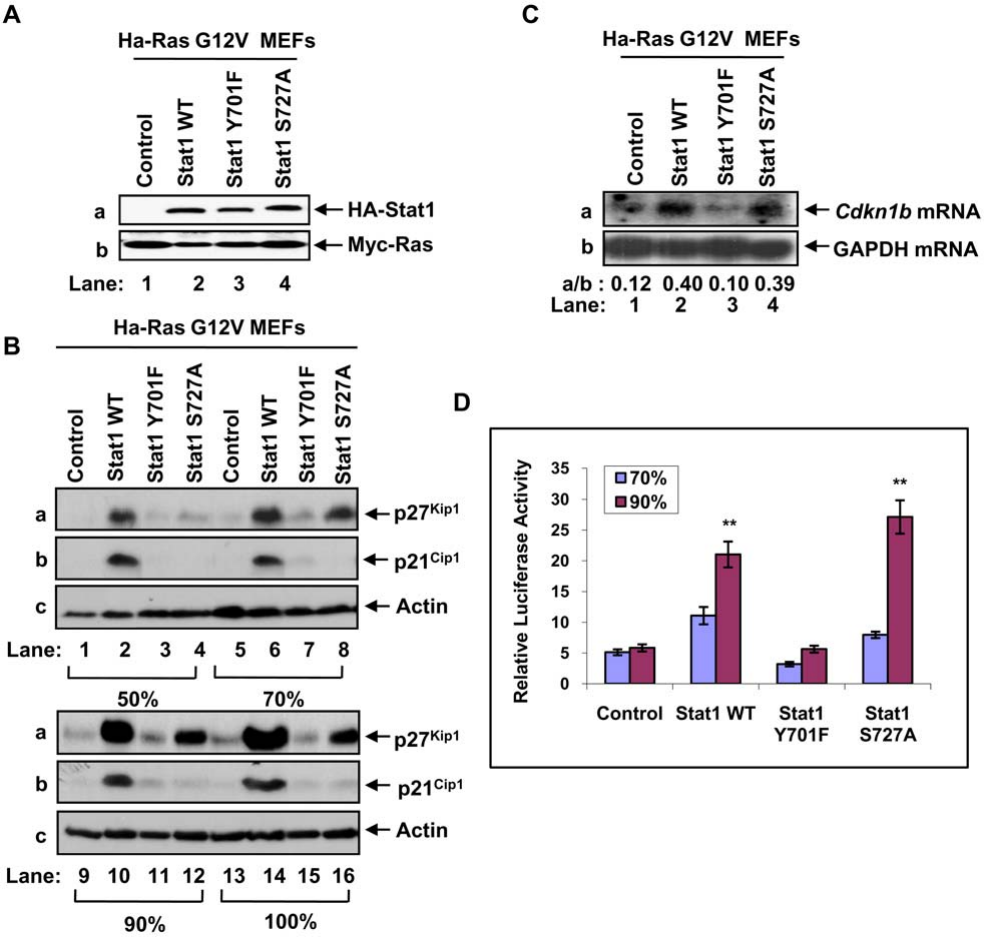
Induction of p27^Kip1^ by Stat1 in Ras-transformed cells depends on site-specific Stat1 phosphorylation. (A) Whole cell extracts (50 µg of protein) from MEFs expressing Myc-Ha-RasG12V and reconstituted with the indicated HA-Stat1 proteins were subjected to immunoblotting with anti-Stat1α mAb (panel a) or anti-Myc mAb (panel b). (B) MEFs were harvested at 50% (lanes 1–4), 70% (lanes 5–8), 90% (lanes 9–12), or 100% confluence (lanes 13–16). Whole cell extracts (50 µg of protein) were subjected to immunoblotting with an anti-p27^Kip1^ mAb (panel a), anti-p21^Cip1^ rabbit polyclonal Ab (panel b) or anti-actin mAb (panel c). (C) Total RNA (15 µg) from MEFs harvested at 90% confluence were subjected to Northern blot analysis using [α-^32^P] dCTP-labeled Cdkn1b cDNA (panel a) and [α-^32^P] dCTP-labelled glyceraldehyde-3-phosphate dehydrogenase (GAPDH, panel b) as probes. The radioactive bands were detected by autoradiography and quantified by densitometry using the NIH Image 1.54 software. (D) Sub-confluent MEFs were transfected with the pGL2 vector containing the firefly luciferase reporter gene under the control of the full-length 1609-bp mouse *Cdkn1b* promoter. Forty eight or 72 hours after transfection, cells at 70% or 90% confluence were harvested and the luciferase activity was determined. The activity of Renilla luciferase expressed from a pGL2 vector lacking the *Cdkn1b* promoter was used as an internal transfection control. Results are expressed ±SD for 3 experiments performed in triplicate. **P<0.01.

Previous findings showed that Stat1 is phosphorylated at Y701 and S727 in Ras transformed cells [27], [28]. To verify these observations, we used NIH3T3 cells transformed with Ha-RasG12V by retrovirus infection. We noted that activated Ras decreased Stat1 Y701 phosphorylation and increased Stat1 S727 phosphorylation compared to control cells in a manner that was dependent upon cell density (Fig. S1). To address the role of Stat1 phosphorylation in p27^Kip1^ expression, the Ras-transfected p53^−/−^Stat1^−/−^ MEFs were reconstituted with HA-Stat1 forms bearing either the Y701F or S727A mutation by retrovirus infection. Immunoblot analysis of the reconstituted MEFs verified that expression of the Stat1 mutants was equal to that of Stat1 WT (Fig. 2A, panel a). When the MEFs were maintained at different levels of confluency, we noted the induction of p27^Kip1^ levels in cells expressing either HA-Stat1 WT or HA-Stat1S727A but not in cells expressing Stat1Y701F or devoid of Stat1 (Fig. 2B, panel a). The induction of p27^Kip1^ was proportional to the increased density of the cells and p27^Kip1^ levels were higher in cells reconstituted with Stat1WT than Stat1S727A (Fig. 2B, panel A). In parallel, we looked at the levels of p21^Cip1^ based on previous findings that Stat1 regulates p21^Cip1^ at the transcriptional level [9] and that p21^Cip1^ levels are upregulated by activated Ras [29]. We found that unlike p27^Kip1^, p21^Cip1^ levels were induced by Stat1 WT only (Fig. 2B, panel b) indicating that p21^Cip1^ expression in Ras transfected cells requires Stat1 phosphorylation at both Y701 and S727.

To address the mechanism of regulation of p27^Kip1^ expression, we first looked at a possible transcriptional effect of Stat1. Northern blot analysis showed an increase in *Cdkn1b* mRNA levels in Ras-transfected MEFs reconstituted with either Stat1 WT or Stat1 S727A compared to control MEFs or MEFs reconstituted with Stat1Y701F (Fig. 2C, panel a). To further substantiate this finding, we assessed the transcriptional activation of the mouse 1.6-Kb *Cdkn1b* promoter by Stat1 in luciferase reporter assays [30]. We found that transcription of the *Cdkn1b* promoter was more highly induced in Ras-transfected MEFs expressing either Stat1 WT or Stat1S727A than in control MEFs or MEFs expressing Stat1Y701F (Fig. 2D). Consistent with the p27^Kip1^ protein levels (Fig. 2B), Stat1-dependent transcription from the mouse *Cdkn1b* promoter was proportional to the increased density of the MEFs (Fig. 2D). These data demonstrated a transcriptional role for Stat1 in *Cdkn1b* gene expression.

### Transcriptional induction of the *Cdkn1b* gene by Stat1 requires Stat3

**Figure 3 pone-0003476-g003:**
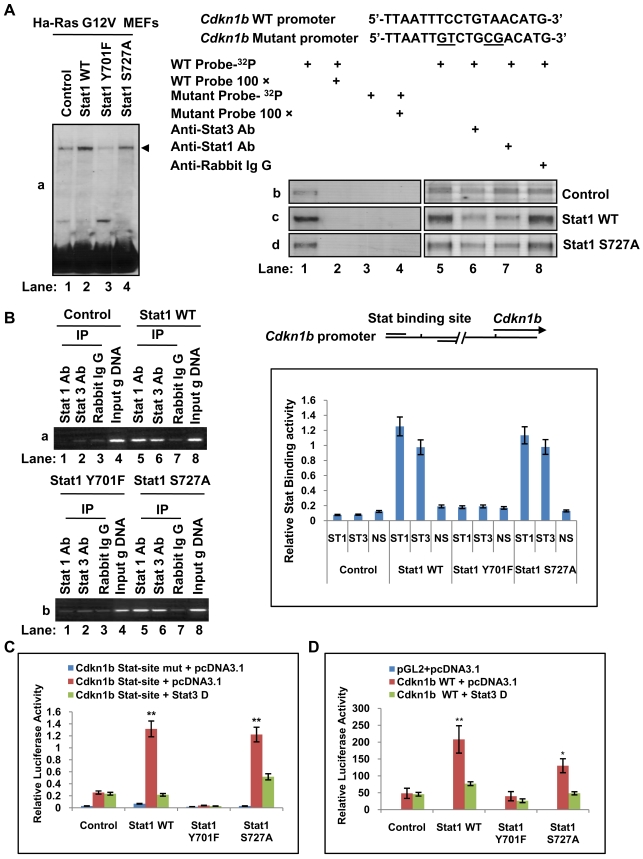
Transcriptional induction of *Cdkn1b* gene by Stat1 requires Stat3. (A) (Left panel) Protein extracts from MEFs harvested at 90% confluence (panel a, lanes 1–4) were subjected to EMSA using a [^32^P]-labelled double-stranded oligonucleotide containing the Stat-binding site within the mouse *Cdkn1b* promoter. (Right panels) The same protein extracts from control MEFs (panel b) and MEFs reconstituted with either Stat1 WT (panel c) or Stat1S727A (panel d) were subjected to EMSAs with various specificity controls as indicated. (B) Detection of Stat1 and Stat3 binding to the *Cdkn1b* promoter by ChIP assays. Detection of *Cdkn1b* promoter DNA after immunoprecipitation with anti-Stat1, anti-Stat3 or rabbit IgG antibodies was performed by PCR. Primers were designed to amplify a 201 bp fragment containing the Stat-binding site of the promoter as indicated. Input gDNA refers to PCR amplification of the 201 bp fragment from genomic DNA purified from each type of MEFs. Quantification of Stat1 and Stat3 binding from 3 independent experiments is shown (graph in blue). (C and D) MEFs were transfected with either the pGL3 vector containing the firefly luciferase gene under the control of three tandem repeats of Stat-binding sites of the *Cdkn1b* promoter (C) or the pGL2 vector containing the firefly luciferase reporter gene under the control of the full-length 1609-bp mouse *Cdkn1b* promoter (D). As control, the same pGL3 vector with mutations in the Stat binding sites was used (C). Transfections included the Stat3-D cDNA expressed from the pcDNA3 vector. Firefly luciferase activity was measured 48 hours in confluent (C) or sub-confluent cells (D). The firefly luciferase levels were normalized to Renilla luciferase driven from the minimal promoter in the pGL3 vector utilized as an internal control. Results are expressed ±SD for 3 experiments performed in triplicate. ** P<0.01.

The mouse *Cdkn1b* promoter contains a Stat-binding site located at a position 1585 bp upstream of the transcription initiation site [18], [31]. To assess the role of the Stat-binding site in *Cdkn1b* transcription by Stat1, we performed electrophoretic mobility shift assays (EMSAs) using extracts from MEFs containing activated Ras and reconstituted with the various forms of Stat1, and a probe encompassing the Stat-binding site of the *Cdkn1b* promoter [18]. To increase the detection of DNA-binding, EMSAs were performed with protein extracts from 90% confluent cells in which *Cdkn1b* promoter activity was maximal (Fig. 2D). We detected the formation of a high molecular weight protein/DNA complex, whose intensity was enhanced in MEFs reconstituted with either HA-Stat1 WT or HA-Stat1 S727A (Fig. 3A, left panel, a). The formation of the complex was abolished when EMSAs were performed in the presence of a 100 fold excess of non-radioactive oligonucleotide (Fig. 3A, right panels b, c and d, lane 2) or when an oligonucleotide with mutations in the Stat-binding site was used as a probe (right panels b, c and d, lanes 3 and 4). To identify the proteins that form the complex, we performed the assay in the presence of antibodies against Stat1 or Stat3. We observed that formation of the complex in MEFs devoid of Stat1 was decreased by 50% after incubation with an antibody against Stat3 (right panel b, compare line 5 with 6) indicating the presence of Stat3 in the complex. Contrary to this, incubation with an antibody against Stat1 or rabbit IgG antibody, which served as a negative control, did not affect binding strength (Fig. 3A, right panel b, lanes 6 and 7). When the EMSAs were performed with protein extracts from MEFs reconstituted with either Stat1 WT (panel c) or Stat1S727A (panel d), we noted that the formation of the complex was decreased after incubation with antibodies against Stat3 (lane 6) or Stat1 (lane 7) but not after incubation with the rabbit IgG antibody (lane 8). The reduction but not elimination of complex formation after incubation with antibodies against Stat1 or Stat3 is consistent with a previous study showing that antibodies against Stat1 or Stat3 did not abolish binding of the Stat1/Stat3 complex to the Stat-site of the *Cdkn1b* promoter in mouse 32D lymphoid cells stimulated with G-CSF [18]. Nevertheless, the possibility remains that the Stat-site is also occupied by a third protein, which forms a complex with DNA and migrates with the same mobility as the Stat1/Stat3 complex in the polyacrylamide gels.

To confirm the DNA-binding data, we employed chromatin immunoprecipitation (ChIP) assays to assess binding of Stat1 and Stat3 to the *Cdkn1b* promoter *in vivo* (Fig. 3B). We detected the specific binding of Stat1 and Stat3 to the promoter in MEFs reconstituted with either Stat1 WT (panel a, lanes 5 and 6) or Stat1S727A (panel b, lanes 5 and 6). On the other hand, the intensity of Stat1 or Stat3 binding was equal to the intensity of binding detected after immunoprecipitation with irrelevant IgG in control MEFs (panel a, lane 2) as well as in MEFs reconstituted with Stat1Y701F (panel b, lanes 1 and 2). These data suggest a weak binding of Stat3 to the promoter DNA in the absence of Stat1 or in the presence of non tyrosine phosphorylated Stat1. Collectively, the above data showed that both Stat1 and Stat3 are bound to the Stat-binding site in the *Cdkn1b* promoter in a manner that is dependent on Y701 phosphorylation of Stat1.

We further examined the effect of Stat1 and Stat3 on the transcriptional activation of the *Cdkn1b* promoter. To this end, we employed a vector containing a luciferase reporter gene under the control of three tandem repeats of the Stat-binding site from the *Cdkn1b* promoter [18]. As control, the same vector containing three tandem repeats of a mutant form of the Stat-binding site was used [18]. We found that luciferase expression from the wild type Stat-binding site was significantly induced in Ras-transfected MEFs that were reconstituted with either Stat1 WT or Stat1 S727A compared to control MEFs or MEFs expressing the Stat1Y701F (Fig. 3C). Interestingly, co-expression of Stat3-D, a Stat3 mutant defective in transactivation activity that exerts a dominant negative effect [32], impaired luciferase expression in MEFs reconstituted with either Stat1 WT or Stat1S727A (Fig. 3C). The role of Stat3 was further verified in transient transfections of the Ras-transfected MEFs with a luciferase reporter gene under the control of the 1.6-Kb mouse *Cdkn1b* promoter (Fig. 3D). We found that the induction of expression of the reporter gene by Stat1 WT or Stat1S727A was blocked by the co-expression of Stat3-D mutant (Fig. 3D). Collectively, these data suggested that Stat1-dependent *Cdkn1b* gene expression requires the activity of Stat3.

### p27^Kip1^ contributes to the inhibition of cell cycle progression by Stat1

**Figure 4 pone-0003476-g004:**
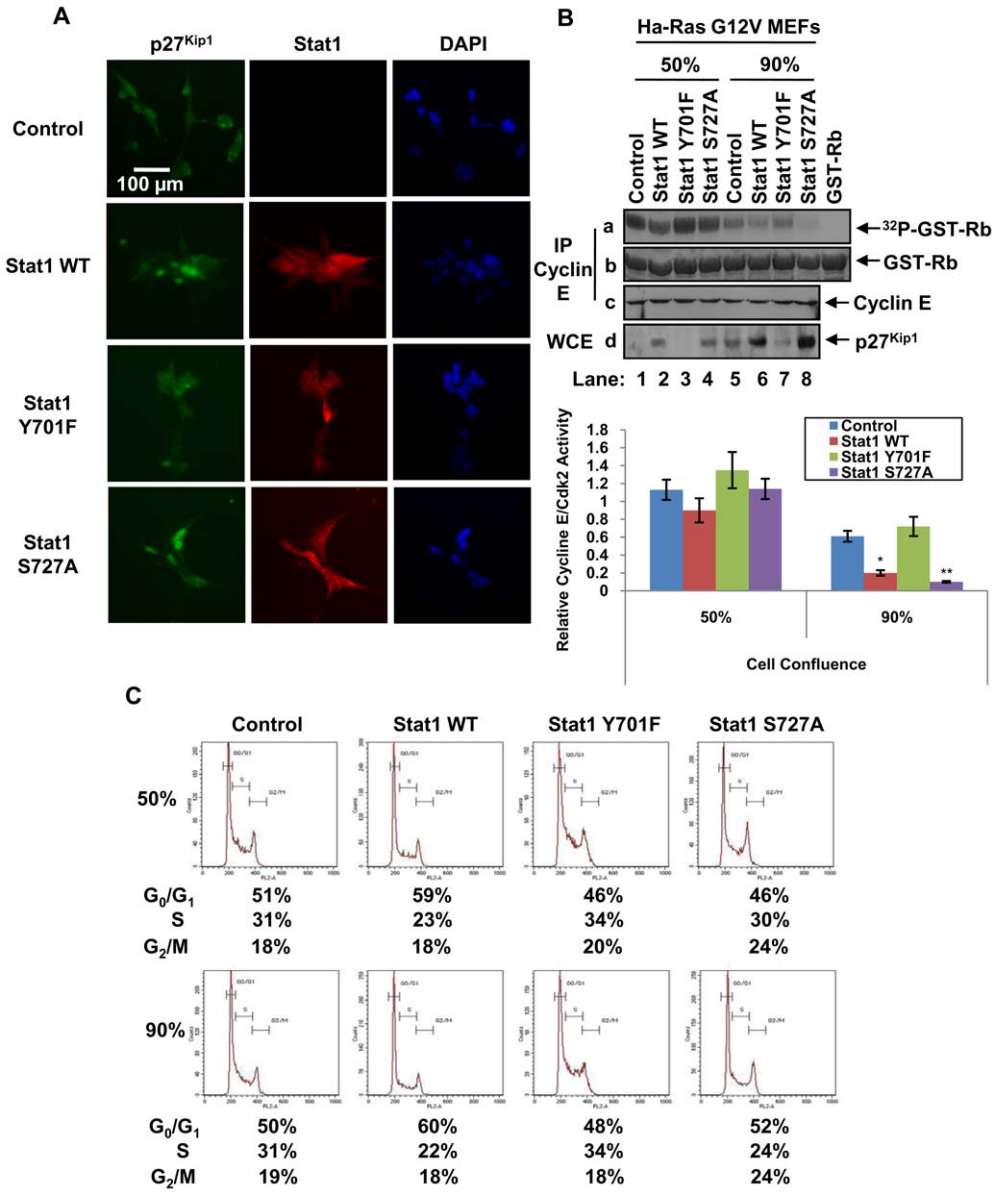
p27^Kip1^ contributes to the inhibition of cell cycle progression by Stat1. (A) MEFs maintained at 70% confluence were subjected to immunostaining with an anti-p27^Kip1^ mAb and a goat anti-mouse IgG conjugated to Alexa Fluro 488 (green). The nucleus was visualized by 4,6-diamidino-2-phenylindole (DAPI) staining. (B) Protein extracts (500 µg) from MEFs that reached 50% (lanes 1–4) or 90% confluence (lanes 5–8) were subjected to immunoprecipitation with an anti-Cyclin E antibody followed by *in vitro* kinase assays using GST-Rb (1 µg) and 1 µCi of [γ-^32^P] ATP (panel a). GST-Rb protein levels were visualized by Commassie blue staining (panel b). The levels of Cyclin E (panel c) and p27^Kip1^ (panel d) in the kinase assays were detected by immunoblotting. CyclinE-Cdk2 activity was assessed by normalizing GST-Rb phosphorylation levels to GST-Rb protein levels. The graph shows results expressed as ±SD from 3 independent experiments (*P<0.05; **P<0.01). (C) Cells were harvested at 50% (upper panel) or 90% confluence (lower panel), stained with propidium iodide and analyzed for DNA content by flow cytometry. The data shown represent one out of three reproducible experiments.

To better understand the biological significance of our findings, we looked at the localization of p27^Kip1^ in Ras-transfected MEFs. Nuclear localization of p27^Kip1^ is required for inhibition of cell cycle progression, which is counteracted in Ras transformed cells by the increased nucleocytoplasmic export and proteasomal degradation of p27^Kip1^ [12]. We found that p27^Kip1^ was both cytoplasmic and nuclear in MEFs lacking Stat1 (control cells) as well as in MEFs reconstituted with Stat1Y701F (Fig. 4A). However, p27^Kip1^ was predominantly nuclear in MEFs expressing either Stat1 WT or Stat1S727A compared to control MEFs or MEFs containing Stat1Y701F (Fig. 4A). On the other hand, Stat1 and its phosphorylation mutants were both nuclear and cytoplasmic in the reconstituted Ras-transfected MEFs (Fig. 4A). To determine whether increased p27^Kip1^ expression (Fig. 2B) had an effect on cell cycle progression, we measured the activity of the Cyclin E-Cdk2 complex, which is predominantly targeted by p27^Kip1^ [12]. To this end, Cyclin E-Cdk2 was purified by immunoprecipitation and subjected to *in vitro* kinase assays using GST-retinoblastoma (Rb) as substrate [33]. We found that Cyclin E-Cdk2 activity was reduced in Ras-transfected MEFs containing either Stat1 WT or Stat1 S727A (Fig. 4B, panel a). Although Cyclin E-Cdk2 activity declined with the increased density of all MEFs, the ability of either Stat1 WT or Stat1S727A to further inhibit CyclinE-Cdk2 activity was still evident in cells maintained at high confluence (Fig. 4B, see quantification in right panel). When we examined cell cycle progression by flow cytometry, we observed a blockade at G_0_/G_1_ phase in MEFs reconstituted with Stat1 WT when cells were maintained at either low or high confluence (Fig. 4C). Inhibition of cell cycle progression was also observed in confluent MEFs expressing Stat1S727A although to a lesser extent than in Stat1 WT MEFs (Fig. 4C). This difference between Stat1 WT and Stat1S727A can be explained by the ability of Stat1WT to upregulate both p21^Cip1^ and p27^Kip1^ and inhibit multiple Cyclin-Cdk complexes (Fig. 2B). These findings provided evidence that p27^Kip1^ expression contributes to the inhibition of cell cycle progression by Stat1 in a manner that is dependent on Stat1 phosphorylation and cell density.

### Phosphorylated Stat1 inhibits Ras-mediated transformation

**Figure 5 pone-0003476-g005:**
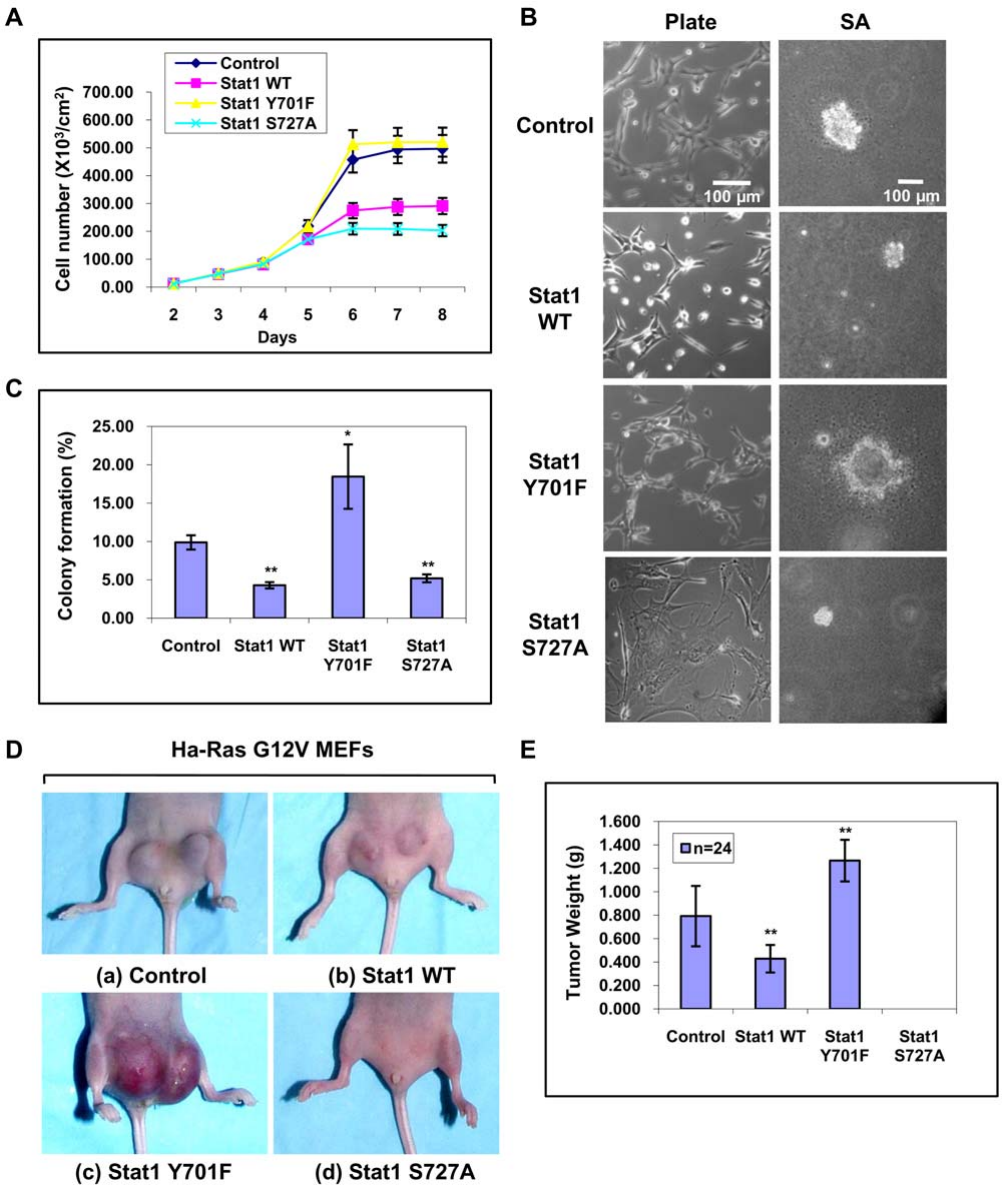
Stat1 inhibits Ras-mediated transformation. (A) Growth rates of the MEFs were determined by counting the number of cells for the indicated periods of time. The results represent ±SD from two reproducible experiments performed in duplicate. (B) Morphological characteristics of MEFs grown on tissue culture dishes (left panel) or in soft agar (SA) (right panel). Bar, 100 µm. (C) Ras-transfected MEFs reconstituted with various Stat1 were plated in soft agar and let grow for 2 weeks. Colony formation of MEFs in soft agar was evaluated for clones larger than 100 µm. Data shown are ±SD from three independent experiments. *P<0.05; ** P<0.01. (D) MEFs were injected into 12 female athymic nude mice (Balb/c nu/nu). Each mouse received two subcutaneous injections (1×10^6^ cells for each of the 2 sites of injection) in the abdominal area proximal to the rear limbs (n = 2×12 = 24 injections). Mice were observed for tumor formation for ∼3 weeks until the largest tumor size became 2 cm in size at which point animals were sacrificed and tumors were excised and weighed. (E) Statistical analysis of tumor formation at 3 weeks post-injection. The average tumor weight (g) and ±SD are shown. **P<0.01.

Next we examined the transforming potential of the Ras-transfected MEFs expressing various forms of Stat1. First, we observed that all MEFs propagated at similar rates when they were maintained at sub-confluent levels. However, in cells at high density, we observed a significant (50%) inhibition in the proliferation of MEFs reconstituted with either Stat1 WT or Stat1 S727A (Fig. 5A). Morphologically, Stat1 WT-expressing MEFs exhibited altered adhesive/spreading properties compared to control MEFs lacking Stat1 or MEFs reconstituted with each of the Stat1 phosphorylation mutants (Fig. 5A, left panels). When we looked at the growth of these cells in soft agar, we observed significant differences (Fig. 5B, right panels). Specifically, Ras-transfected MEFs reconstituted with either Stat1 WT or Stat1 S727A formed fewer colonies than control MEFs or MEFs reconstituted with Stat1Y701F (Fig. 5B, right panels). Furthermore, Ras-transfected MEFs with either Stat1 WT or Stat1 S727A yielded colonies that were smaller in size by 80% than the colonies derived from the control MEFs. On the other hand, MEFs reconstituted with Stat1 Y701F produced both the highest number of colonies and the largest colonies (Fig. 5C). Decreased colony formation by Stat1 WT or Stat1 S727A was not due to induction of Stat1-dependent apoptosis [34] as verified by annexin V staining and FACS analysis (Fig. S2). The differences in soft agar growth prompted us to examine the tumorigenic potential of the Ras-transfected MEFs. Tumor growth was assessed by the subcutaneous injection of the cells in athymic nude mice (Balb/c nu/nu). We found that control MEFs produced larger tumors than Stat1WT MEFs, which yielded ∼50% smaller tumors (Figs. 5D and E). On the other hand, MEFs reconstituted with Stat1Y701F yielded the largest tumors of all (Figs. 5D and E). Interestingly, MEFs expressing Stat1S727A did not produce tumors within the 3 week observation period (Figs. 5D and E) but yielded detectable tumors (∼2 mm) approximately 2.5 months after injection (data not shown). Histochemical analysis indicated that all tumors were high grade soft tissue sarcomas (data not shown). These data demonstrated that Stat1 functions as a suppressor of Ras-mediated oncogenesis in a manner that is dependent on site-specific Stat1 phosphorylation.

### p27^Kip1^ contributes to the inhibition of Ras-mediated transformation by Stat1

**Figure 6 pone-0003476-g006:**
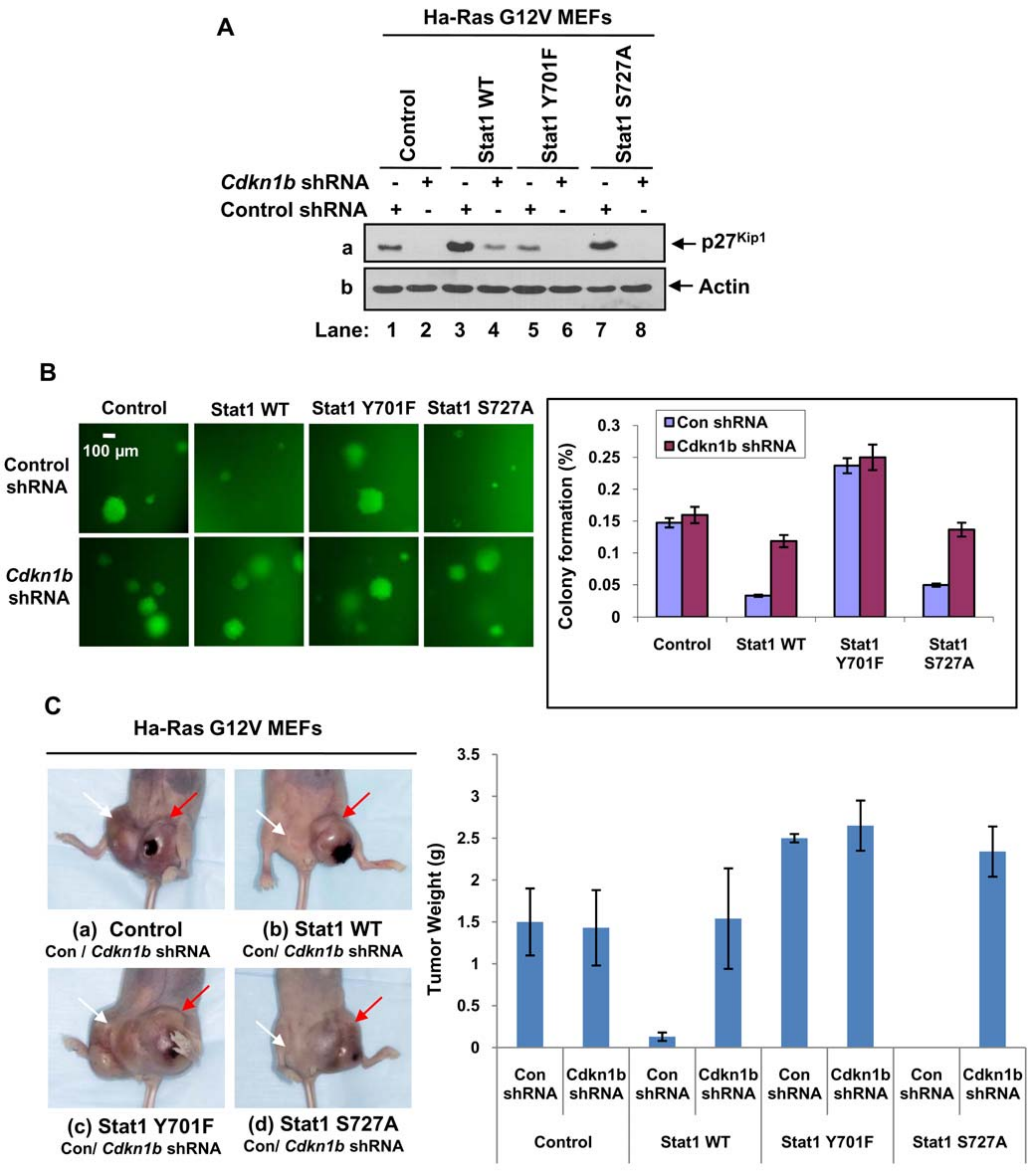
p27^Kip1^ mediates the inhibition of Ras-mediated transformation by Stat1. (A) MEFs were infected with retroviruses bearing a shRNA against luciferase reporter gene (control shRNA) or shRNA against mouse *Cdkn1b* mRNA. Protein extracts (50 µg) were subjected to immunoblot analysis for p27^Kip1^ (panel a) and actin (panel b). (B) Cells expressing control shRNA or *Cdkn1b* shRNA were plated in soft agar and let grow for 2 weeks. Colonies expressing GFP (green) were evaluated for their ability to grow larger than 100 µm in size. Data shown are ±SD from three independent experiments. Colony formation (%) represents the number of cells forming colonies larger than 100 µm out of hundred plated cells. *P<0.05; **P<0.01. (C) MEFs treated with control shRNA or *Cdkn1b* shRNA were injected into 3 female athymic nude mice (Balb/c nu/nu). Each mouse received two subcutaneous injections (1×10^6^ cells per site of injection) in the abdomen proximal to the rear limbs. One injection contained MEFs treated with control shRNA (left side, white arrow) and the other injection contained MEFs treated with *Cdkn1b* shRNA (right side, red arrow). Mice were observed for tumor formation for ∼3 weeks until the largest tumor size became 2 cm in size at which point animals were sacrificed and tumors were excised and weighed. Statistical analysis of tumor formation at 3 weeks post-injection is shown in the graph. The average tumor weight (g) and ±SD are indicated. **P<0.01.

Previous findings established an essential role of p27^Kip1^ in the inhibition of Ras-mediated tumorigenesis [35]. To determine whether inhibition of Ras transformation by Stat1 involves p27^Kip1^, we assessed the transforming activity of the MEFs when endogenous p27^Kip1^ levels were decreased by shRNA. To this end, knockdown of p27^Kip1^ was achieved by infection of Ras transfected MEFs with retroviruses bearing *Cdkn1b* shRNA and the green fluorescence protein (GFP) as a marker [36]. As control, retroviruses bearing GFP and a shRNA against the luciferase reporter gene were used [36]. Decrease of p27^Kip1^ in the shRNA-treated MEFs was verified by immunoblotting (Fig. 6A). When the cells were plated in soft agar, we observed that anchorage-independent growth was restored in MEFs reconstituted with either Stat1 WT or Stat1S727A in which p27^Kip1^ was targeted by shRNA as indicated by the growth of the GFP-positive (green) colonies (Fig. 6B). On the other hand, decreased p27^Kip1^ levels did not further increase the ability of control MEFs lacking Stat1 or MEFs reconstituted with Stat1Y701F to form colonies in soft agar (Fig. 6B). The role of p27^Kip1^ in the inhibition of Ras-mediated tumorigenesis by Stat1 was further evaluated in nude mice. That is, tumor growth after subcutaneous injection of the MEFs in nude mice was significantly enhanced for MEFs reconstituted with either Stat1 WT or Stat1S727A and treated with shRNA against p27^Kip1^ (Fig. 6C). Contrary to this, downregulation of p27^Kip1^ in control MEFs or MEFs reconstituted with Stat1Y701 did not further enhance tumor growth (Fig. 6C). These finding demonstrated a major role of p27^Kip1^ in the inhibition of Ras-mediated oncogenesis by Stat1.

**Figure 7:**
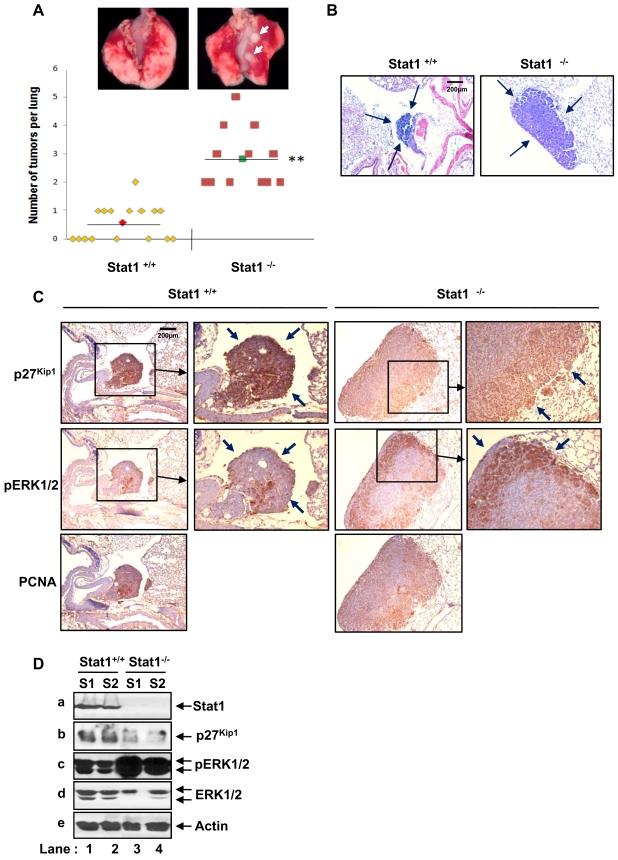
Stat1 inhibits lung tumor formation by activated K-Ras. (A) Lungs were dissected and tumors (indicated by arrows) were counted by visual inspection 28 weeks after urethane treatment. Tumor multiplicity was increased in Stat1^−/−^ mice (n = 12) compared to Stat1^+/+^ mice (n = 15). ** P<0.01. (B) Eosin and hematoxylin staining of lungs tissues from Stat1^+/+^ and Stat1^−/−^ mice. The location of tumors in the stained lung tissue is indicated by arrows. Tumors were classified as bronchioalveolar adenomas and papillary adenomas. (C) Lung tissue from urethane-treated Stat1^+/+^ or Stat1^−/−^ mice was subjected to immunohistochemical analysis for p27^Kip1^(top panels), phospho-(p)Erk1/2 (middle panels) and proliferating cell nuclear antigen (PCNA) (bottom panels). Staining of the areas in rectangles is shown in higher magnification in the right panels. The location of tumors is indicated by arrows. (D) Protein extracts (50 µg) from Stat1^+/+^(T1, T2) and Stat1^−/−^(T3, T4) lung tumors were separated by SDS-PAGE and subjected to immunoblot analysis for Stat1, p27^Kip1^, phospho(p)-Erk1/2, total Erk1/2 and actin.

To further substantiate the importance of p27^Kip1^ and Stat1 in the suppression of Ras transformation, we examined the susceptibility of Stat1^+/+^ and Stat1^−/−^ mice to urethane-induced tumorigenesis. Specifically, urethane treatment results primarily in the development of lung tumors that carry an activating mutation at codon 61 of K-Ras [37], [38]. Loss of p27^Kip1^ was shown to significantly increase the incidence and growth of lung tumors of mice treated urethane [39]. When Stat1^+/+^ and Stat1^−/−^ mice were treated with a single intraperitoneal injection of urethane, we observed the development of tumors in both animal groups 28 weeks after treatment (Fig. 7A). However, only 50% of the animals in the Stat1^+/+^ group (8 out of 16) developed small tumors (<0.7 mm) as opposed to the Stat1^−/−^ group in which all animals (n = 12) developed large tumors (>2 mm). Histological analysis indicated that the lung tumors were a mixture of bronchioalveolar adenomas and papillary adenomas (Fig. 7B). Immunohistochemical analysis further showed a high amount of p27^Kip1^ in lung tumors from Stat1^+/+^ mice compared to lung tumors from Stat1^−/−^ mice (Fig. 7C). Consistent with tumor growth, we detected a higher amount of phosphorylated Erk1/2 in Stat1^−/−^ than in Stat1^+/+^ lung tumors, which indicated the induction of the Ras-MAPK pathway by activated K-Ras (Fig. 7C). Interestingly, Erk1/2 phosphorylation levels were inversely proportional to p27^Kip1^ levels in the lung tumors as detected by immunohistochemistry (Fig. 7C) and immunoblotting (Fig. 7D). These data further indicated that both p27^Kip1^ and Stat1 function together to suppress Ras-mediated oncogenesis *in vivo*.

## Discussion

Our findings uncover an important function of Stat1 in the regulation of p27^Kip1^ with implications in Ras-mediated tumorigenesis. The ability of Stat1 to act upstream of p27^Kip1^ is a property of Ras transformation because Stat1 did not exhibit similar effects on *Cdkn1b* gene transcription or expression and localization of p27^Kip1^ in immortalized MEFs (Fig. S3). Moreover, induction of *Cdkn1b* gene transcription by Stat1 in Ras-transformed MEFs is independent of p53 (Fig. S4). The transcriptional effect of Stat1 on the *Cdkn1b* promoter depends on Y701 phosphorylation but is independent of S727 phosphorylation. Given the essential role of S727 phosphorylation in the transactivation properties of Stat1 in response to IFNs [40], [41], the dispensable role of S727 phosphorylation in the induction of the *Cdkn1b* gene indicated that transactivation of the *Cdkn1b* promoter was mediated by a protein other than Stat1. Consistent with this notion, our data demonstrated that the transcriptional induction of the *Cdkn1b* gene by Stat1 occurs in cooperation with Stat3. Stat3 was previously shown to activate the *Cdkn1b* gene during myeloid cell differentiation in response to IL-6 and G-CSF [18], [42]. The ability of Stat1 to cooperate with Stat3 in the transcriptional activation of the *Cdkn1b* gene suggests that Stat1 is capable of re-programming the biological function of Stat3 by converting it from a positive to a negative regulator of cell proliferation. At first glance, the ability of Stat3 to induce the expression of p27^Kip1^ in Ras transformed cells was not in line with its well characterized role as a positive regulator of cell proliferation and tumorigenesis [43], [44]. However, recent findings support the notion that Stat3 also possesses the capacity to impair cell proliferation and oncogenesis in a manner that depends on the signalling pathway and the genetic background of the target cells [45]. Although transcriptional control of the *Cdkn1b* gene by Stat1 plays a major role in regulating p27^Kip1^ levels in Ras transformed cells, the possibility that Stat1 can also regulate *Cdkn1b* gene expression at the post-transcriptional level can not be ruled out. This notion is supported by the observation that p27^Kip1^ is more highly expressed in Ras transformed MEFs reconstituted with Stat1 WT than with Stat1S727A (Fig. 2B) although both Stat1 proteins induce *Cdkn1b* gene transcription at comparable levels (Figs. 2C and D). Possible post-transcriptional regulation of *Cdkn1b* gene expression may occur at the level of mRNA translation and/or protein stability. At the translational level, Stat1 was previously shown to signal to the cellular translational machinery via physical and functional interactions with the eIF2α kinase PKR [46], [47]. At the post-translational level, the potential effects of Stat1 may be exerted through its ability to inhibit the transcription of *c-myc* [48], which induces the expression of proteins, including Cyclin D1, that sequester and inhibit p27^Kip1^ [13]. This is consistent with previous findings showing that Stat1 impairs *c-myc* and induces p27^Kip1^ expression in human monocytic U-937 cells in response to all-trans retinoid acid [49].

The anti-proliferative effects of Stat1 in response to IFNs are partly mediated by its ability to inhibit cell cycle progression [9], [48]. Our findings show that Stat1 is required for the upregulation of both p21^Cip1^ and p27^Kip1^ in Ras transformed cells in the absence of IFN treatment. It has been well documented that mitogens increase p21^Cip1^ levels through the activation of Ras and Raf-MAPK signalling [29] which results in increased transcription of the *Cdkn1a* gene [50]. Consistent with these findings, we found that induction of p21^Cip1^ in Ras transformed cells is dependent on Stat1 and is mediated at the transcriptional level (data not shown). However, unlike p27^Kip1^, the induction of p21^Cip1^ levels in Ras transformed cells depends on both tyrosine and serine phosphorylation of Stat1. Although Stat1 upregulates p21^Cip1^ and p27^Kip1^ levels in Ras transformed cells through separate mechanisms, both Cdk inhibitors appear to be involved in G_0_/G_1_ arrest (Fig. 4). It is of interest that the cell cycle inhibitory effects of Stat1 were increased in confluent cell cultures indicating a role of intercellular adhesion signalling in this process. Consistent with this observation, it was shown that Stat1 becomes activated by the focal adhesion kinase (FAK) with important implications in the regulation of cell adhesion and migration [51]. Given that p27^Kip1^ plays an important role in cell motility independent of its cell cycle regulatory functions [52], regulation of p27^Kip1^ levels by Stat1 may also have profound roles in cell migration and tumor metastasis [53].

Several findings support the anti-tumor function of Stat1 [43], [44]. Specifically, Stat1^−/−^mice are more prone to chemical induced carcinogenesis than Stat1^+/+^ mice, and Stat1^−/−^ mice bred onto p53^−/−^ background develop spontaneous tumors more rapidly than the p53^−/−^ mice [6]. The high incidence of tumor formation in Stat1^−/−^ animals is partly explained by impaired tumor immunosurveillance caused by defects in IFN-γ-signalling and natural killer cell activity [3]. Previous work established that the sensitivity of tumors to IFN-γ is required for the development of an anti-tumor response in immunocompetent hosts [6]. Because nude mice are not completely immunodeficient [54], the observed differences in tumor growth of the Ras transformed MEFs might have been attributed to their responsiveness to IFN-γ. However, we found that the responsiveness of the Ras transformed MEFs to IFN-γ did not correlate with their growth properties in nude mice. That is, although IFN-γ-mediated gene transactivation was impaired in Ras transformed cells expressing Stat1S727A (Fig. S5), these cells were barely tumorigenic in nude mice (Fig. 5). These observations argued against a role of tumor immunosurveillance in regulation of tumor growth in nude mice in our system. Also, growth of Ras-transformed cells in soft agar correlated with their growth in nude mice (Fig. 5) further supporting a direct role of Stat1 in suppression of Ras-mediated tumorigenesis. Our approach with shRNA clearly demonstrated that the anti-tumor activity of Stat1 is dependent on p27^Kip1^ (Fig. 6). It is of interest that tumor growth of Ras-transformed MEFs in nude mice is more highly suppressed by Stat1S727A than Stat1 WT (Fig. 5). Inasmuch as both Stat1 [53] and p27^Kip1^ [55] are involved in suppression of angiogenesis and Stat1 phosphorylation is affected by tumor hypoxia [56], tumor microenvironment may have more pronounced effects on the inhibition of tumor growth of Ras-transformed cells containing Stat1S727A than cells containing Stat1 WT. The effects of Stat1 are not confined to Ras-transformed MEFs only since activation of the K-Ras pathway in lung tissue by urethane results in a higher tumor incidence in Stat1^−/−^ than in Stat1^+/+^ mice (Fig. 7). Although the increased tumor formation in urethane treated Stat1^−/−^ mice could partly involve defects in tumor immunosurveillance [54], the higher incidence of lung tumor formation in Stat1^−/−^ compared to Stat1^+/+^ mice was proportional to Ras-MAPK activation and inversely proportional to p27^Kip1^. Given that urethane-treated *Cdkn1b* ^−/−^ mice were more prone to lung tumorigenesis than *Cdkn1b* ^+/+^ mice [57], together these data suggest that Stat1 and p27^Kip1^ act in the same pathway to inhibit Ras-mediated oncogenesis. It is of interest that ERK1/2 phosphorylation was diminished in lung tumors containing Stat1 compared to tumors devoid of Stat1 (Fig. 7C and D). This result indicated that Stat1 may have an inhibitory effect on Ras-MAPK signalling. Consistent with this notion, we noted that reconstitution of Ras-transfected Stat1^−/−^p53^−/−^ MEFs with Stat1 WT resulted in a significant inhibition of ERK1/2 phosphorylation compared to control MEFs or MEFs reconstituted with each of the Stat1 phosphorylation mutants (Fig. S6). The molecular basis of this inhibition is not immediately clear and represents the focus of future experiments. Given that activation of the Ras-MAPK pathway results in the degradation of p27^Kip1^ [13], inhibition of the Ras-MAPK pathway by Stat1 may also reveal its ability to regulate p27^Kip1^ at the post-translational level.

There has been an established link between Stat1 phosphorylation and human cancer [44]. Specifically, Stat1 is constitutively phosphorylated at Y701 in many blood tumors including multiple myeloma, erythroleukemia and acute myelogenous leukemia (AML) [58]. In the case of solid tumors, Y701 phosphorylation of Stat1 has been detected in breast as well as in head and neck cancers [44]. Moreover, S727 phosphorylation of Stat1 is induced in chronic lymphocytic leukemia (CLL) [58], in Wilms' tumors [59] as well as in tumor cells deficient in tuberous sclerosis complex (TSC) 1 and 2 [60]. Given that phosphorylation is essential for Stat1 activation, detection of phosphorylated Stat1 in human tumors appears to be inconsistent with its anti-proliferative and tumor suppressor activities. However, recent findings showed that the anti-tumor function of Stat1 is determined by the type of the tumor and the oncogenic signalling within it. That is, Stat1 was shown to act as a promoter of leukemogenesis induced by v-abl and TEL-Jak2 oncogenes [61]. Our findings suggest a different regulation of the anti-tumor activity of Stat1 by site-specific phosphorylation. That is, activated Ras has the capacity to decrease Y701 phosphorylation and increase S727 phosphorylation of Stat1 (Fig. S1). These differences in Stat1 phosphorylation may significantly contribute to Ras-mediated tumorigenesis based on the ability of Stat1S727A or Stat1Y701F to compromise or promote the transforming activity of Ras in MEFs respectively (Figs. 5, 6). As such, it is reasonable to speculate that differences in the equilibrium between serine and tyrosine phosphorylation of Stat1 could determine the outcome of an oncogenic insult and the efficacy of chemotherapies aimed at inducing Stat1 phosphorylation [62]–[65]. Although Stat1 phosphorylation mutants have not been identified in human cancers, our findings indicate that Stat1 phosphorylation in tumors may interfere with the normal function of Stat1 and that the occurrence and frequency of site-specific phosphorylated Stat1 in human cancers could be of significant diagnostic and prognostic value. Consistent with this notion, tyrosine phosphorylation of Stat1 was shown to be a marker in the prognostic evaluation of breast tumors [66] as well as of head and neck tumors [67].

## Materials and Methods

### Animals and treatments

BALB/c Stat1^−/−^ mice [4] and wild type BALB/c mice from Harlan labs were maintained as previously described [4]. Athymic mice (Balb/c nu/nu), female and 4–6 weeks old, were provided by Charles River. Urethane treatment was carried out using a previously described protocol [57]. Mice were sacrificed after 28 weeks, dissected and examined for lung tumors. Lungs were fixed in formalin, embedded in paraffin, and slides (4 µm thick) were subjected to immuno-histochemical analysis. Tumors in athymic mice were monitored daily for ∼3 weeks to ensure that the conditions and good welfare of the animals were not compromised. The mice were sacrificed when the tumor size reached 2 cm at which point they became cumbersome or necrotic. The animal experiments were performed in accordance with approved protocols and regulations by the Animal Welfare Committee of McGill University (protocol #3271).

### Cell culture procedures

Mouse embryonic fibroblasts (MEFs) and NIH3T3 cells (ATCC CRL-1658) were maintained in Dulbecco's modified Eagle's medium (DMEM) (Gibco) supplemented with 10% calf serum and antibiotics. Infection with pBabe-expressing retroviruses was described elsewhere [47]. The luciferase assays were performed with the Dual-Luciferase Reporter Assay System (Promega) using *Renilla* luciferase as an internal control. Soft agar growth assays were performed as described [68].

### Plasmids and antibodies

Myc-tagged Ha-RasG12V cDNA was subcloned into the EcoRV site of pcDNA3.1/Hygro (Invitrogen). The pBabe vector containing wild type (WT) HA-Stat1 was described previously [47]. HA-Stat1S727A was produced by the QuickChange site-directed mutagenesis (Stratagene) using the primers 5′- CAACCTGCTCCCCATGGCACCTGAGGAGTTTGACGAGG-3′ and 5′-CCTCGTCA AACTCCTCAGGTGCCATGGGGAGCAGGTTG-3′ on wild type template vector. HA-Stat1S727A and HA-Stat1Y701F cDNA [69] were subcloned into the SnaB I site of the pBabe vector. *Cdkn1b* shRNA and luciferase shRNA in a pSIREN vector were reported elsewhere [36]. The pGL2 vector containing the luciferase reporter gene under the control of full length mouse *Cdkn1b* promoter (−1609 to + 178 bp) was described [30]. The PGL3 vectors containing the luciferase gene under the control of wild type or mutant Stat-binding site of the *Cdkn1b* promoter was described [18]. The Stat3-D cDNA in pcDNA expression vector was previously described [32].

Anti-Stat1α p91(C-111), anti-Myc (9E10), anti-Stat3(C-20), anti-Stat1(M-23) and anti-p21(C-19) antibodies were purchased from Santa Cruz Biotechnology; anti-pY701-Stat1, anti-pS727-Stat1 and phosphor-p44/p42 MAPK (Thr202/Tyr204) antibodies from Cell Signalling; anti-actin (C4) from Biosource International; anti-p27^Kip1^ antibody from BD Transduction Laboratories; anti-Cyclin E rabbit serum was provided by Dr. A. Besson. The horseradish peroxidase (HRP)-conjugated anti-mouse IgG antibody and HRP anti-rabbit IgG antibody were from Amersham Pharmacia Biotech. The Alexa Fluor 488 conjugated goat anti-mouse IgG and Alexa Fluor 546 conjugated goat anti-rabbit IgG antibodies were from Molecular Probes.

### Immunoblottings, immunoprecipitations, immunofluorescence and flow cytometry

Immunoblottings and immunoprecipitations were performed as described [70] whereas cell cycle analysis was based on a established protocol [71]. Immunofluorescence analysis was performed as reported [72].

### Northern blotting and electrophoretic mobility shift assays (EMSAs)

Northern blotting using 15 µg of total RNA was performed as described [70]. EMSAs were performed based on a previously established protocol [46] using an oligonucleotide encompassing the Stat-binding site of the mouse *Cdkn1b* promoter in wild type form (5′-TTAATTTCCTGTAACATG-3′) or in its mutant form (5′- TTAATTGTCTGCGACATG-3′; mutations are underlined) as reported [18].

### Chromatin immunoprecipitation (ChIP) assays

ChIP assays were carried out based on a protocol described elsewhere [73]. Polymerase chain reaction (PCR) was performed using the *Cdkn1b* forward primer 5′-GTGGCTAAGAAAACAAGTCAAT-3′ and reverse primer 5′-TAGCCAGGCCTGTCGTATCTCA-3′. The conditions were: 94°C for 5 min, 30 cycles at 94°C for 1 min, 55°C for 1 min, 72°C for 30 sec and a final elongation at 72°C for 10 min.

### Cyclin E –Cdk2 kinase assay

Immunoprecipitation of Cyclin E-Cdk2 and *in vitro* kinase assays using GST-Rb were performed as previously described [33].

## Supporting Information

Figure S1 *Control of Stat1 phosphorylation by activated Ras.* NIH3T3 cells were infected with pBabe retroviruses lacking (control; Con) or bearing activated Ha-RasG12V. After selection in 2 µg/ml puromycin for 2 weeks, polyclonal populations were maintained at different levels of confluency (50–100%). Protein extracts (50 µg) were subjected to immunoblot analysis for Stat1 phosphorylated at Y701 (panel a) or S727 (panel b), total Stat1 (panel c), ERK1/2 phosphorylated at Thr202/Tyr204 (panel d), total ERK1/2 (panel e) or actin (panel f). The doublet recognized by the Stat1 Y701 phosphospecific antibody most likely represents the two isoforms (α and β) of Stat1.
(6.40 MB TIF)

Figure S2 *Evaluation of apoptosis in Ras-transformed Stat1^−/−^p53^−/−^ MEFs reconstituted with the various forms of Stat1.* Sub-confluent Ras-transformed Stat1^−/−^p53^−/−^ MEFs lacking (Control) or reconstituted with either Stat1 WT or Stat1S727A were subjected to staining with Annexin V-propidium iodide (PI) staining according to the manufacturer's specifications (Biosource). Cells were then subjected to flow cytometry analysis by using FACScan (Becton Dickinson), and data were analyzed by using WinMDI version 2.8 software (The Scripps Institute). The data represent one out of two reproducible experiments.
(4.84 MB TIF)

Figure S3 *Detection of p27Kip1 localization and Cdkn1b mRNA levels in immortalized MEFs containing Stat1 WT or Stat1 phosphorylation mutants.* (A) Spontaneously immortalized isogenic Stat1^−/−^ MEFs as well as Stat1^−/−^ MEFs reconstituted with Stat1 WT were subjected to immunostaining for endogenous p27^Kip1^ and Stat1 as described in Fig. 4A. (B) Immortalized Stat1^−/−^ MEFs reconstituted with either Stat1 WT or Stat1 phosphorylation mutants (i.e. Stat1Y701F, Stat1S727A) were maintained at 90% confluency and subjected to Northern blot analysis for detection of endogenous *Cdkn1b* (a) and GAPDH mRNA levels (b) as described in Fig. 2C. The levels of reconstituted Stat1 proteins were detected by immunoblot analysis (panel c). The data represent one out of two reproducible experiments.
(5.93 MB TIF)

Figure S4 *Examination of the role of p53 in induction of Cdkn1b gene transcription by Stat1.* Ras-transformed Stat1^−/−^p53^−/−^ MEFs (Control) and Ras-trasnformed Stat1^−/−^p53^−/−^ MEFs reconstituted with Stat1 WT (Stat1 WT) were transfected with pCL2 vector containing the firefly luciferase reporter gene under the control of the full length mouse *Cdkn1b* promoter (Cdkn1bWT) together with the pcDNA3.0 vector lacking (pcDNA3) or containing the mouse wild type p53 cDNA (p53). As control, pCL2 vector containing the firefly luciferase gene but lacking the *Cdkn1b* promoter was used. The firefly luciferase levels were normalized to Renilla luciferase driven from the minimal promoter in the pGL3 vector used as an internal control. Results are expressed ±SD for 3 experiments performed in triplicate.
(3.95 MB TIF)

Figure S5 *Control of IFN-γ-mediated gene transactivation in Ras-trasnfected MEFs.* MEFs were transiently transfected with a firefly luciferase reporter gene under the control of a promoter containing two IFN-γ-activated sites (GAS) from the IFP53 gene (pGL-2XIFP53 GAS luciferase). Thirty two hours post transfection cells were left untreated or treated with 500 IU/ml of mouse IFN-γ (Biosource) for 12 hours. Cells were harvested and assayed for firefly luciferase activity and normalized to an internal control consisting of a renilla luciferase reporter. Results are expressed ±SD for 3 experiments performed in triplicate.
(3.37 MB TIF)

Figure S6 *Detection of ERK1/2 phosphorylation in Ras-transfromed MEFs.* Protein extracts (50 µg) from confluent cells were subjected to immunoblotting for ERK1/2 phosphorylated at Thr202/Tyr204 (panel a) as well as for total ERK1/2 (panel b). The ratio of phosphorylated to non-phosphorylated ERK1/2 for each lane is indicated. The data represent one out of two reproducible experiments.
(3.77 MB TIF)
