# Supplementary figures and images for: Stat1 Phosphorylation Determines Ras Oncogenicity by Regulating p27Kip1
Source: PLoS One. 2008 Oct 22;3(10):e3476. doi: 10.1371/journal.pone.0003476 (PMC2568943; doi:10.1371/journal.pone.0003476)

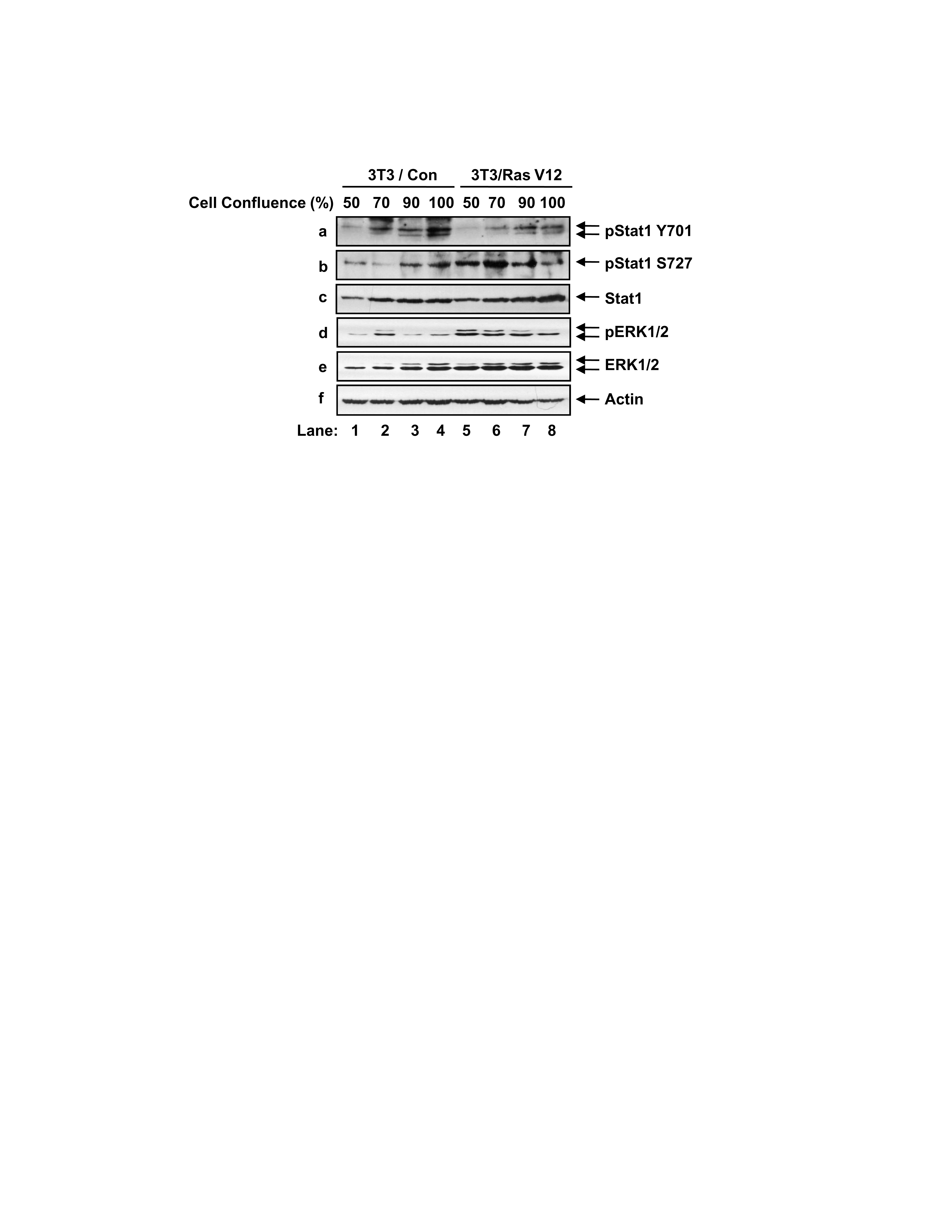

Supplement: Figure S1 — Control of Stat1 phosphorylation by activated Ras. NIH3T3 cells were infected with pBabe retroviruses lacking (control; Con) or bearing activated Ha-RasG12V. After selection in 2 µg/ml puromycin for 2 weeks, polyclonal populations were maintained at different levels of confluency (50–100%). Protein extracts (50 µg) were subjected to immunoblot analysis for Stat1 phosphorylated at Y701 (panel a) or S727 (panel b), total Stat1 (panel c), ERK1/2 phosphorylated at Thr202/Tyr204 (panel d), total ERK1/2 (panel e) or actin (panel f). The doublet recognized by the Stat1 Y701 phosphospecific antibody most likely represents the two isoforms (α and β) of Stat1. (6.40 MB TIF) [file pone.0003476.s001.tif]

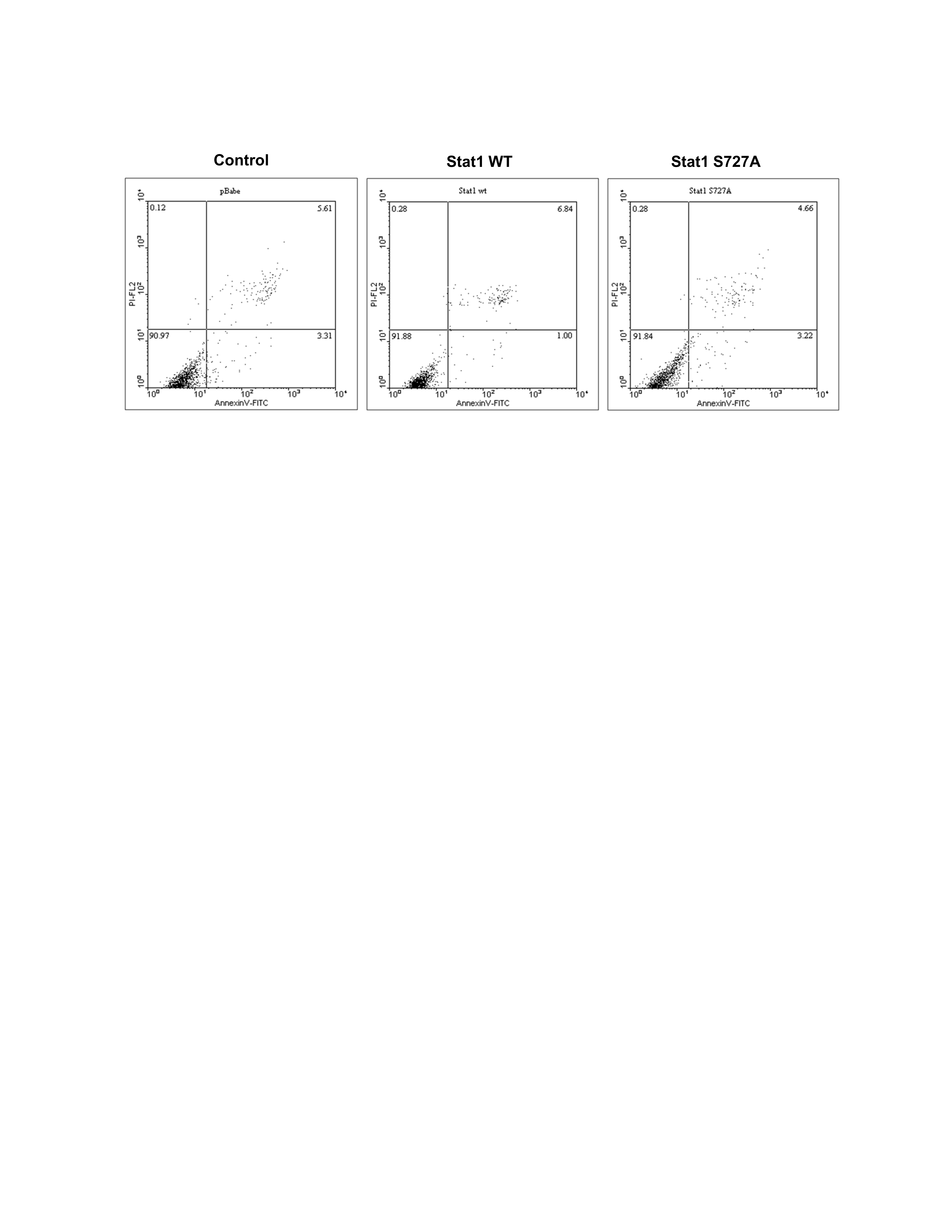

Supplement: Figure S2 — Evaluation of apoptosis in Ras-transformed Stat1−/−p53−/− MEFs reconstituted with the various forms of Stat1. Sub-confluent Ras-transformed Stat1−/−p53−/− MEFs lacking (Control) or reconstituted with either Stat1 WT or Stat1S727A were subjected to staining with Annexin V-propidium iodide (PI) staining according to the manufacturer's specifications (Biosource). Cells were then subjected to flow cytometry analysis by using FACScan (Becton Dickinson), and data were analyzed by using WinMDI version 2.8 software (The Scripps Institute). The data represent one out of two reproducible experiments. (4.84 MB TIF) [file pone.0003476.s002.tif]

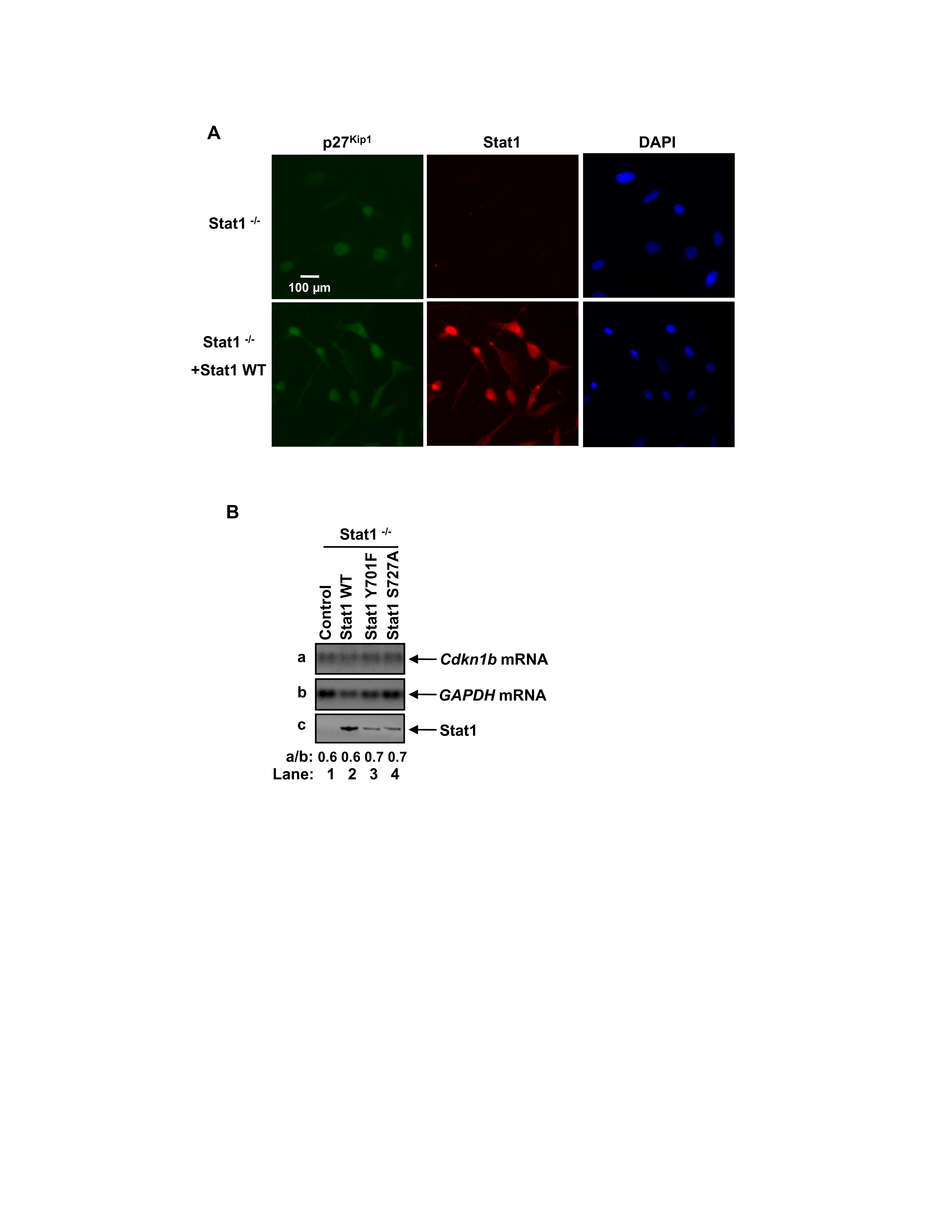

Supplement: Figure S3 — Detection of p27Kip1 localization and Cdkn1b mRNA levels in immortalized MEFs containing Stat1 WT or Stat1 phosphorylation mutants. (A) Spontaneously immortalized isogenic Stat1−/− MEFs as well as Stat1−/− MEFs reconstituted with Stat1 WT were subjected to immunostaining for endogenous p27Kip1 and Stat1 as described in Fig. 4A. (B) Immortalized Stat1−/− MEFs reconstituted with either Stat1 WT or Stat1 phosphorylation mutants (i.e. Stat1Y701F, Stat1S727A) were maintained at 90% confluency and subjected to Northern blot analysis for detection of endogenous Cdkn1b (a) and GAPDH mRNA levels (b) as described in Fig. 2C. The levels of reconstituted Stat1 proteins were detected by immunoblot analysis (panel c). The data represent one out of two reproducible experiments. (5.93 MB TIF) [file pone.0003476.s003.tif]

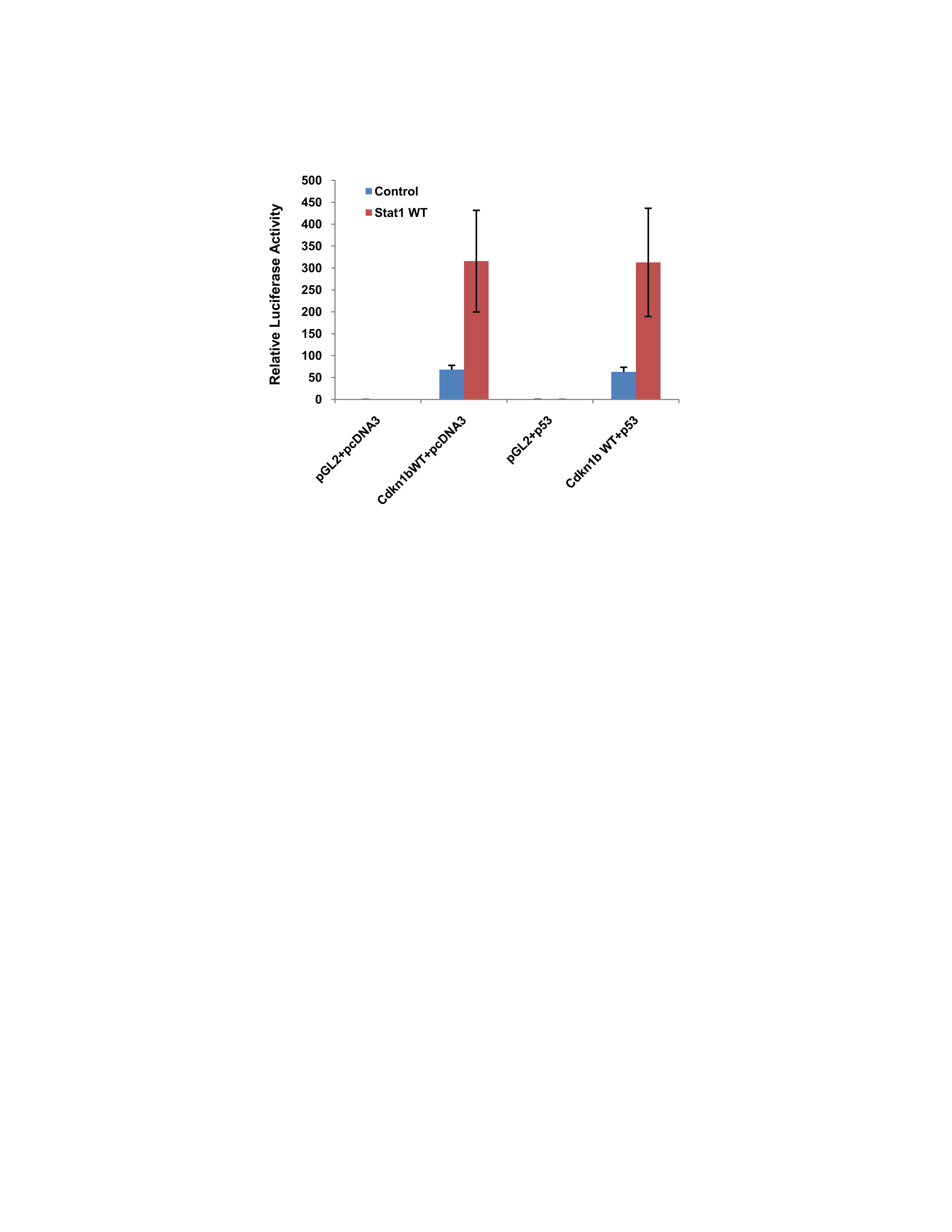

Supplement: Figure S4 — Examination of the role of p53 in induction of Cdkn1b gene transcription by Stat1. Ras-transformed Stat1−/−p53−/− MEFs (Control) and Ras-trasnformed Stat1−/−p53−/− MEFs reconstituted with Stat1 WT (Stat1 WT) were transfected with pCL2 vector containing the firefly luciferase reporter gene under the control of the full length mouse Cdkn1b promoter (Cdkn1bWT) together with the pcDNA3.0 vector lacking (pcDNA3) or containing the mouse wild type p53 cDNA (p53). As control, pCL2 vector containing the firefly luciferase gene but lacking the Cdkn1b promoter was used. The firefly luciferase levels were normalized to Renilla luciferase driven from the minimal promoter in the pGL3 vector used as an internal control. Results are expressed ±SD for 3 experiments performed in triplicate. (3.95 MB TIF) [file pone.0003476.s004.tif]

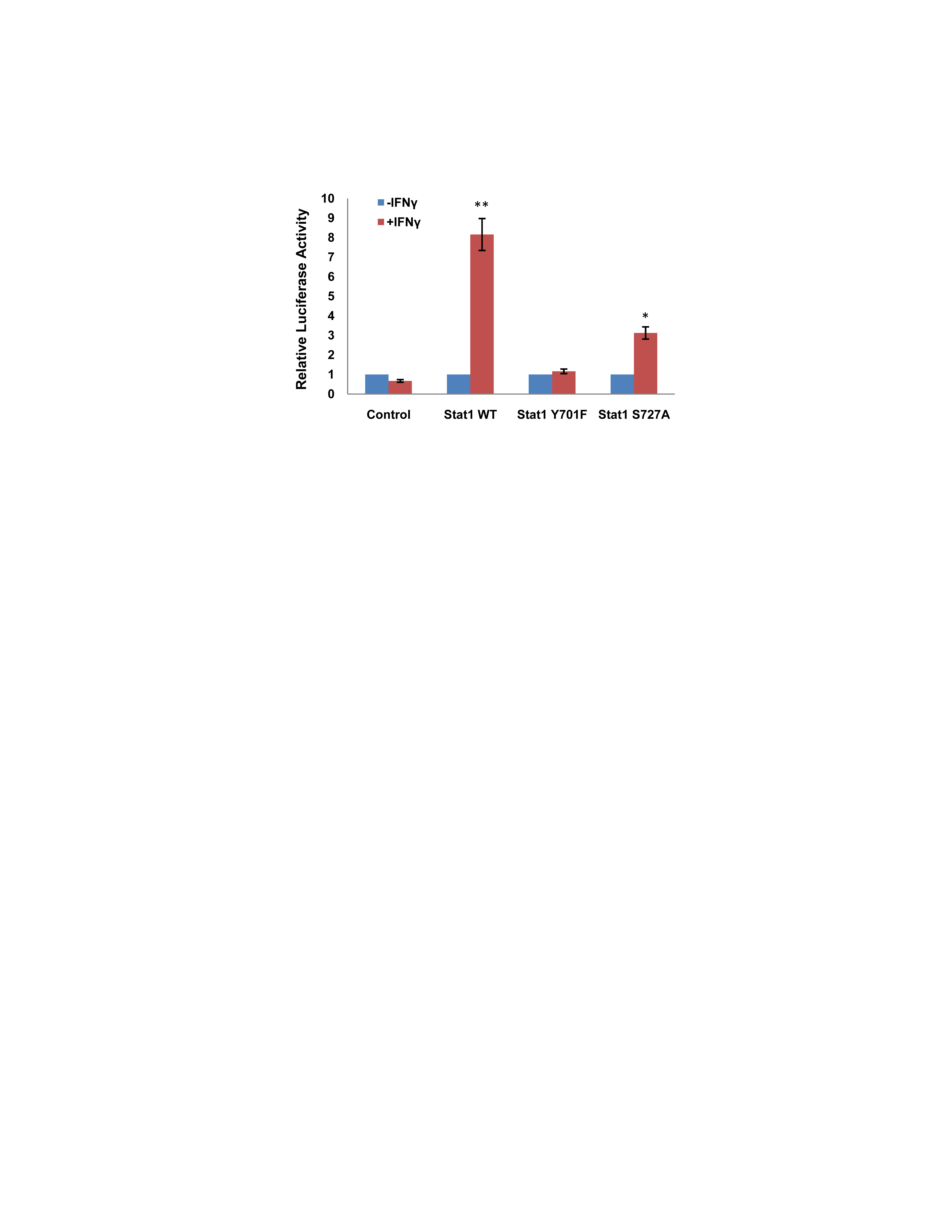

Supplement: Figure S5 — Control of IFN-γ-mediated gene transactivation in Ras-trasnfected MEFs. MEFs were transiently transfected with a firefly luciferase reporter gene under the control of a promoter containing two IFN-γ-activated sites (GAS) from the IFP53 gene (pGL-2XIFP53 GAS luciferase). Thirty two hours post transfection cells were left untreated or treated with 500 IU/ml of mouse IFN-γ (Biosource) for 12 hours. Cells were harvested and assayed for firefly luciferase activity and normalized to an internal control consisting of a renilla luciferase reporter. Results are expressed ±SD for 3 experiments performed in triplicate. (3.37 MB TIF) [file pone.0003476.s005.tif]

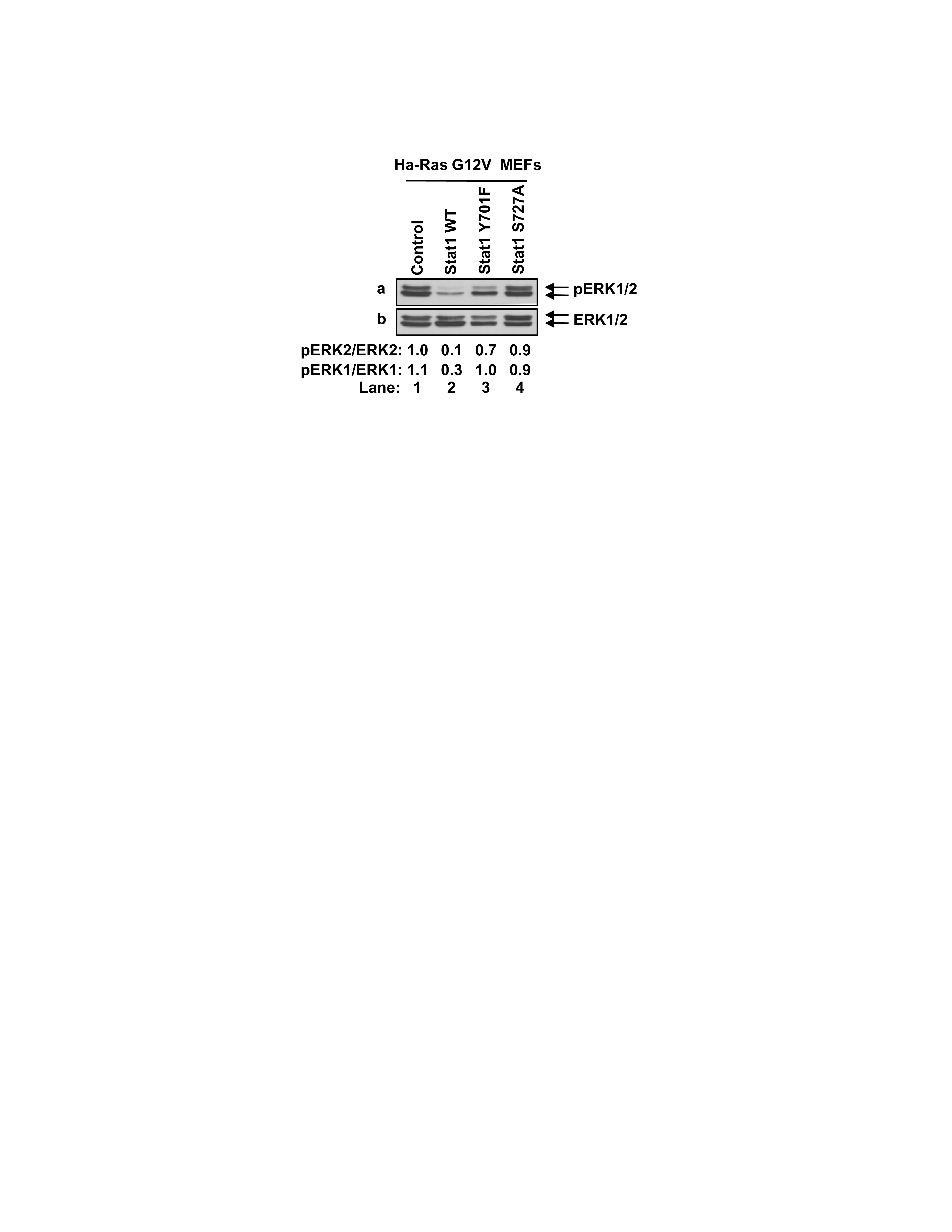

Supplement: Figure S6 — Detection of ERK1/2 phosphorylation in Ras-transfromed MEFs. Protein extracts (50 µg) from confluent cells were subjected to immunoblotting for ERK1/2 phosphorylated at Thr202/Tyr204 (panel a) as well as for total ERK1/2 (panel b). The ratio of phosphorylated to non-phosphorylated ERK1/2 for each lane is indicated. The data represent one out of two reproducible experiments. (3.77 MB TIF) [file pone.0003476.s006.tif]
